# Association of childhood pulmonary tuberculosis with exposure to indoor air pollution: a case control study

**DOI:** 10.1186/s12889-019-6604-9

**Published:** 2019-03-07

**Authors:** Nkosana Jafta, Prakash M. Jeena, Lars Barregard, Rajen N. Naidoo

**Affiliations:** 1 Discipline of Occupational and Environmental Health, School of Nursing and Public Health, University of KwaZulu-Natal, 321 George Campbell Building, Howard College Campus, Durban, 4041 South Africa; 2 Discipline of Pediatrics and Child Health, School of Clinical Medicine, University of KwaZulu-Natal, Private Bag X1, Congella, Durban, 4013 South Africa; 3 Department of Occupational and Environmental Medicine, Sahlgrenska University Hospital and Sahlgrenska Academy at Gothenburg University, Box 414, S-405 30 Gothenburg, Sweden

**Keywords:** Indoor air pollution, Childhood tuberculosis, Dampness, Secondhand smoke, Exposure assessment, Risk factors

## Abstract

**Background:**

Crude measures of exposure to indicate indoor air pollution have been associated with the increased risk for acquiring tuberculosis. Our study aimed to determine an association between childhood pulmonary tuberculosis (PTB) and exposure to indoor air pollution (IAP), based on crude exposure predictors and directly sampled and modelled pollutant concentrations.

**Methods:**

In this case control study, children diagnosed with PTB were compared to children without PTB. Questionnaires about children’s health; and house characteristics and activities (including household air pollution) and secondhand smoke (SHS) exposure were administered to caregivers of participants. A subset of the participants’ homes was sampled for measurements of PM_10_ over a 24-h period (*n* = 105), and NO_2_ over a period of 2 to 3 weeks (*n* = 82). IAP concentrations of PM_10_ and NO_2_ were estimated in the remaining homes using predictive models. Logistic regression was used to look for association between IAP concentrations, crude measures of IAP, and PTB.

**Results:**

Of the 234 participants, 107 were cases and 127 were controls. Pollutants concentrations (μg/m^3^) for were PM_10_ median: 48 (range: 6.6–241) and NO_2_ median: 16.7 (range: 4.5–55). Day-to-day variability within- household was large. In multivariate models adjusted for age, sex, socioeconomic status, TB contact and HIV status, the crude exposure measures of pollution viz. cooking fuel type (clean or dirty fuel) and SHS showed positive non-significant associations with PTB. Presence of dampness in the household was a significant risk factor for childhood TB acquisition with aOR of 2.4 (95% CI: 1.1–5.0). The crude exposure predictors of indoor air pollution are less influenced by day-to-day variability. No risk was observed between pollutant concentrations and PTB in children for PM_10_ and NO_2_.

**Conclusion:**

Our study suggests increased risk of childhood tuberculosis disease when children are exposed to SHS, dirty cooking fuel, and dampness in their homes. Yet, HIV status, age and TB contact are the most important risk factors of childhood PTB in this population.

## Background

In 2016 tuberculosis (TB) is one of the leading causes of morbidity and mortality in the world with a rate of 140 (95% Confidence Interval (CI), 915–1150) per 100,000 people and 22 (95% CI, 21–24) per 100,000 people respectively [1]. Of the 10.4 million annual TB cases reported globally in 2016, 10–11% occur in children [1, 2] with developing countries having the biggest burden of this disease [1]. In South Africa, the overall reported incidence of TB has been constantly decreasing from a rate of 977 (95% CI, 911–1030) per 100,000 in 2007 to 860 (95% CI, 776–980) per 100,000 in 2013 [3]. In 2016, South Africa was one of top six countries with highest burden of TB disease in the world with an incidence rate of 781 (95% CI, 543–1060) cases per 100,000 people [1]. The reported number of adults and children with coinfection of TB and HIV has stayed around 60% [4].

Environmental risk factors associated with acquisition of tuberculosis include exposure to indoor air pollution (IAP) and secondhand smoke (SHS) with these factors having a higher impact on children compared to adults [5, 6]. A meta-analysis by Patra et al. [6] showed an association between secondhand smoke (SHS) exposure and risk of active TB in children (pooled Odds Ratio (OR) = 3.4, 95% CI: 1.8–6.4) to be higher than in the adult population (pooled OR = 1.3,(95% CI: 1.0–1.7). Associations between exposure to IAP or SHS and TB have been estimated using crude measures of exposure to indoor air pollution, such as fuel type used in cooking and heating and the number of smokers in the house. None of the indoor air studies measured or estimated indoor pollutant concentrations, while some studies measuring outdoor air pollution looked at the relationship between air pollution and tuberculosis [7–9]. The objective of this study was to determine associations between indoor air pollution in the homes of children and pulmonary tuberculosis disease in Durban, South Africa.

## Methods

The study was conducted in Durban, which is the third biggest city in South Africa with a population size estimation of 3.7 million in 2018. This city is located in the province of KwaZulu-Natal in the east coast of the country [10]. Compared to other provinces in the country KwaZulu-Natal has been having high HIV/TB co-infection rate of 64% as well as TB case notification of 678 per 100,000 (60%/100,000) in 2015 [11].

This study employed a case-control design and the target population were children aged 0–14 years of age living in households situated in the Durban metropolitan area. The study period was from May 2011 to November 2014. Caregivers were interviewed about the children’s health using standardized and validated child health questionnaire designed for this study. Environmental assessment of the households was performed with a walkthrough checklist and monitoring of indoor air pollutant concentrations.

**Fig. 1:**
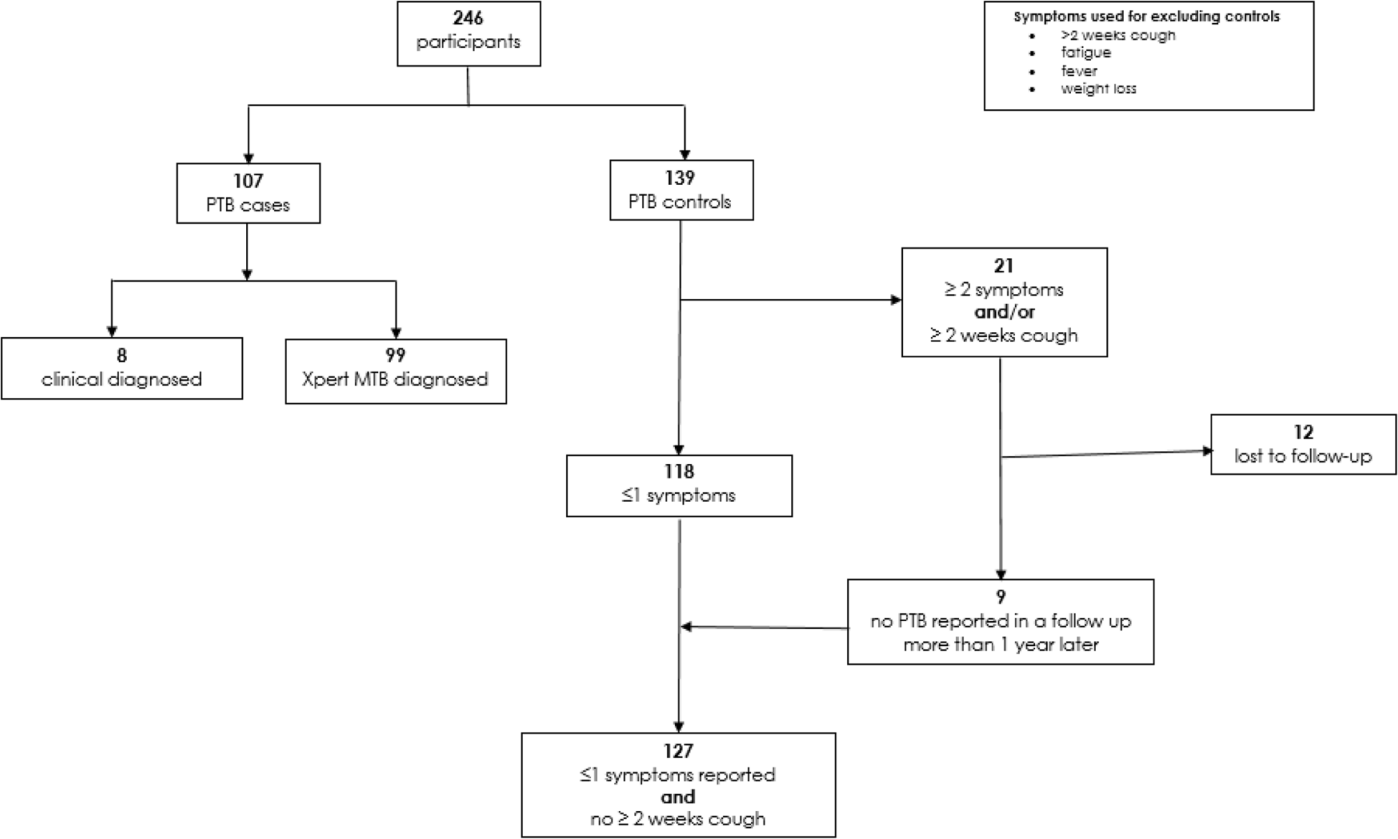
Identification of participants and the definitions of pulmonary tuberculosis cases and controls used in the studies

PTB cases were identified through the diagnosing laboratory or in hospital wards and clinics. The central Prince Mshiyeni Memorial Hospital (PMMH) TB diagnosis laboratory, that tests specimen sent from three (3) district hospitals, namely King Edward VIII Hospital, Prince Mshiyeni Memorial Hospital and RK Khan Hospital, and 31 primary health clinics in Durban, were used to identify PTB cases. These hospitals and clinics were also visited to identify cases. Caregivers of the identified children were subsequently contacted for recruitment. Cases were children diagnosed as having PTB using the Xpert MTB/RIF assay (GeneXpert MTB/RIF; Cepheid, Sunnyvale, CA) and/or clinical diagnosis using the South African National TB Management Guidelines (Fig. 1) [12]. The NDoH protocol for clinical diagnosis of childhood PTB is based on clinical signs and symptoms of TB that include more than 2 weeks of cough, fatigue, fever and weigh loss or malnutrition [12].

The controls were children that had not been diagnosed with PTB. Two approaches were used in identification and recruitment of community controls. The first method was asking the caregiver of the recruited case to identify a neighbor whose child was of the same age group and sex as the case and forty-one controls were identified and recruited using this approach. As there was a general reluctance on the part of the “case” caregiver pointing out other potential “control” children, preschool and primary school parents (*n* = 98) from schools in the same neighbourhood as the case, were approached to allow their children to participate in the study. The caregivers of the controls were interviewed using the same instruments as used for the cases.

Screening of potential controls was performed by asking the caregivers about their child’s recent diagnosis of TB by a health professional. Where no reported diagnosis of TB was identified, the caregiver was interviewed using the same child health questionnaire used in cases. The questionnaire had a section that enquires about symptoms that are used for PTB diagnosis in children (cough of more than 2 weeks duration, fatigue, weight loss and fever). All children that were included as controls, but reported more than two symptoms and/or reported a cough for more than two weeks (*n* = 21) were assessed a year later to determine if they developed TB. Twelve of these controls who could not be contacted were excluded, while the remainder presented with no evidence of TB, and were retained for analysis. A detailed description of the selection of cases and controls is shown in Fig. 1.

The researcher interviewed the caregivers about their child’s health using a standardised close-ended child health questionnaire in isiZulu or English. The questions were from different instruments used to investigate respiratory illnesses in general [13] and screen for childhood TB [12]. The questionnaire included information on the child’s and mother’s health and the socioeconomic status of the family unit. Child health data included symptoms related to TB such as night sweats, chronic cough, loss of weight and fatigue, and receipt of anti-tuberculosis therapy, other current and/or previous respiratory diseases, HIV status and associated treatment and the nutritional status. Other questions included any adult(s) diagnosed with TB (current and past), living in the same household as the child. Indicators of socio-economic status were based on educational levels of child’s caregiver, employment status, household income and household crowding.

A previously validated household walkthrough instrument [14] was used by trained observers to collect information on household characteristics. The instrument consisted of documenting observations on the condition of the house and interviewing the caregiver about information on household activities of the occupants. Housing conditions observations included type of house, material used in the construction of roof, walls and floor, presence of windows, presence of opening windows, visible mould growth and dampness or moisture on surfaces. Activity information collected included type of cooking and heating energy sources used, number of tobacco smokers in the home, burning of incense and use of a heater. The cooking and heating fuels used in the households were classified as clean (electricity and liquid petroleum gas (LPG)) and dirty (kerosene and wood) and those households that used a combination of clean and dirty fuels were classified as mixed fuel [14]. Children living in households with at least one smoker present were considered to be exposed to secondhand tobacco smoke (SHS) [14].

Sampling and analysis of pollutants is detailed elsewhere [14]. In brief, indoor particulate matter of diameter less than 10 μm (PM_10_) was sampled over a period of 24 h using battery operated Airmetrics MiniVol samplers with interchangeable impactors (Tisch Environmental, Eugene, OR). Teflon filters with a support ring of 2 .0μm pore size (PALL, Ann Arbor, MI) were used for sampling PM_10_ in the homes. PM_10_ concentrations were determined by gravimetric analysis in a climate-controlled laboratory. Indoor NO_2_ concentrations were monitored in homes using Radiello® passive samplers (Sigma-Aldrich, St Louis, MO) which were placed for a two to three-week period in the same room in which the PM_10_ sampling was performed. The analysis of NO_2_ was performed by an accredited commercial laboratory using inductively coupled plasma mass spectrometry (ICP-MS).

Particulate matter (PM_10_) and NO_2_ concentrations were measured in 105 and 82 homes respectively. Due to limited availability of resources, these pollutant concentrations were estimated using predictive models in remaining unmeasured households (PM_10_: *n* = 129 and NO_2_: *n* = 152). These models were developed using multivariable linear regression with measured PM_10_ and NO_2_ concentrations as dependent variables and home characteristics and occupants’ activities of the measured homes as independent variables [14]. Variables that predicted PM_10_ concentrations were type of housing structure, total number of rooms, type of primary cooking fuel, season and number of household smokers. Nitrogen dioxide (NO_2_) concentrations were predicted by distance from the roadway, type of housing structure, type of primary cooking fuel, burning of incense and season [14]. The resulting predictive models were used to predict pollutant concentrations for the homes not measured using their home characteristics and occupants’ activities. Missing data points of homes that were not sampled were substituted with predicted pollutant concentrations estimated from modeling [14].

All data from completed child health questionnaires, household walkthrough checklists and analysed indoor air sample results were entered into Excel spreadsheets. Data was subject to logic checks to ensure validity and consistency. A validated and complete dataset was exported to STATA/IC version 13 for further analysis. Analysis was based on 107 PTB cases and 127 controls (Fig. 1).

Firstly, differences between cases and controls in frequency of child, maternal and household characteristics were tested using logistic regression. Home characteristics variables that described IAP were cooking fuel type (clean vs mix or dirty cooking fuel), secondhand smoke (no household smoker vs presence of a household smoker/s), dampness (no visible damp surface vs presence of visible damp surface), PM_10_ and NO_2_ concentrations (measured and estimated). Correlation between PM_10_ and NO_2_ concentrations was tested using Spearman correlation test.

Associations between indoor air pollution and PTB in children were then assessed using stepwise multiple variable logistic regression. Five models with each of the different indicators of indoor environment, namely cooking fuel type, SHS, dampness, PM_10_ and NO_2_ concentrations, as independent variables and childhood PTB as dependent variable were conducted. Variables that were significant determinants of childhood PTB in the univariate model or that had been shown to be risk factors in literature were selected for inclusion. A *p*-value of 0.05 and less was used as cut-off for variables to remain in the model. Age, sex and the pollutant of interest were forced into the models even if the p-value was above 0.05. Sensitivity analyses were carried out by modeling only measured concentrations of PM_10_ and NO_2_ excluding estimated concentrations. In each model possible interaction was tested by inclusion of interaction terms such as presence of opening windows in the household, use of stove extractor fan and permitted indoor smoking.

HIV testing was performed in 143 children as part of their clinical management and was routinely undertaken as part of the research. For statistical analysis, we ran models assuming that those with unknown HIV status were negative and then models that excluded those children whose HIV status was unknown.

The study protocol was approved by the Biomedical Research Ethics Committee of University of KwaZulu-Natal (Ref. number: BREC 104/09) and permission was obtained from the health institutions involved in identification and recruitment of participants. On recruitment, the researcher explained the study to the caregivers and written or oral consent obtained from individual caregivers.

## Results

Among the PTB cases, there was an equal male to female ratio, while controls had a slightly preponderance of females (58%). The mean age of the participants was 7.3 (SD: 3.7) years and cases were somewhat younger (Table 1). Of note, markedly fewer controls had HIV testing done (42%) compared to the cases (84%); 49% of PTB cases tested HIV positive compared to 4% of controls. Household TB contact was more common in cases than in controls (63% vs 25%). Caregivers of the cases who had completed high school were few (6%) compared to the caregivers of the controls (15%) (Table 1).

**Table 1:** Univariate analysis of child and maternal characteristics in childhood pulmonary tuberculosis cases and controls

		cases (*n* = 107)	controls (*n* = 127)	
		n (%)	n (%)	Crude OR (95% CI)
Child Characteristics				
Sex				
	Female	55 (51)	74 (58)	
	Male	52 (49)	53 (42)	1.3 (0.8–2.2)
Age				
	≤2	**26 (24)**	**12 (9)**	
	> 2 to ≤15	**81 (76)**	**115 (91)**	**0.3 (0.2–0.7)**
Hospitalization since birth				
	No	**42 (39)**	**99 (78)**	
	Yes	**65 (61)**	**28 (22)**	**5.5 (3.1–9.7)**
HIV test done				
	No	**17 (16)**	**74 (58)**	
	Yes	**90 (84)**	**53 (42)**	**7.4 (4.0–14)**
HIV positive**				
	No	**46 (51)**	**49 (92)**	
	Yes	**44 (49)**	**4 (8)**	**12 (3.9–35)**
ARV treatment before TB diagnosis^#^				
	Yes	23 (52)	1 (25)	
	No	21 (48)	3 (75)	3.3 (0.3–34)
Household TB contact				
	No	**39 (36)**	**95 (75)**	
	Yes	**67 (63)**	**32 (25)**	**5.0 (2.8–8.7)**
Maternal Characteristics				
Caregiver education				
	Post high school	**6 (6)**	**19 (15)**	
	High school + below	**101 (94)**	**108 (85)**	**3.0 (1.1–7.7)**

Significant difference in households with different characteristics for both cases and controls are shown in bold

*ARV* antiretroviral

**n = 143

^#^*n* = 48

Visible dampness on the room surfaces (OR = 1.8, 95% CI: 1.01–3.1), crowding (OR = 1.8, 95% CI: 1.06–3.2) and mixture of biomass and clean fuels for cooking (OR = 2.6, 95% CI: 1.07–6.4) differed significantly between PTB cases and controls (Table 2). Cases were more likely to be exposed to SHS (48% vs 35%) (OR = 1.7, 95% CI: 0.98–2.8), with a trend towards statistical significance (*p* = 0.059).

**Table 2:** Frequency of child, maternal and household characteristics of childhood pulmonary tuberculosis cases and controls

		cases (*n* = 107)	controls (*n* = 127)	Crude OR (*p*-value)
		n (%)	n (%)	
Household Characteristics				
Type of home				
	Formal	71 (66)	97 (76)	
	Informal	36 (34)	30 (24)	1.6 (0.9–2.9)
Total number of rooms in the household				
	≥4	60 (56)	86 (68)	
	1–3	47 (44)	41 (32)	0.6 (0.4–1.04)
Presence of an opening window				
	All or some rooms	95 (89)	116 (91)	
	No room	12 (11)	11 (9)	0.7 (0.3–1.8)
Visible surface water stains/dampness				
	No room	**28 (26)**	**49 (39)**	
	All or some rooms	**79 (74)**	**78 (61)**	**1.8 (1.01–3.1)**
Visible mold growth presence				
	No room	57 (53)	79 (62)	
	All or some rooms	50 (47)	48 (38)	0.1 (0.9–2.4)
Burning of incense				
	No room	63 (59)	76 (60)	
	All or some rooms	44 (41)	51 (40)	1.04 (0.6–1.8)
Primary cooking fuel				
	Clean	**91 (85)**	**119 (94)**	
	Mix + Dirty	**16 (15)**	**8 (6)**	**2.6 (1.1–6.4)**
Secondary cooking fuel				
	None+ Clean	57 (53)	82 (65)	
	Mix + Dirty	50 (47)	45 (35)	1.6 (0.9–2.7)
Secondhand smoke				
	No	56 (52)	82 (65)	
	Yes	51 (48)	45 (35)	1.7 (0.98–2.8)
Crowding (persons per room)				
	**≤2**	**64 (60)**	**93 (73)**	
	**> 2**	**43 (40)**	**34 (27)**	**1.8 (1.1–3.2)**

Significant difference in households with different characteristics for both cases and controls are shown in bold

The overall median PM_10_ and NO_2_ concentrations from the sampling program was 43 μg/m^3^ (range: 6.6–223) and 16 μg/m^3^ (range: 4.5–55) respectively; with both pollutants having a right-skewed distribution (Table 3). These two pollutants were weakly correlated (r_s_ = 0.30, *p* < 0.001). Although there was a marginally higher median PM_10_ (48 μg/m^3^) among cases when compared to control (41 μg/m^3^), this was not statistically significant. No statistically significant differences were seen for NO_2_ concentrations between cases (median: 15 μg/m^3^) and controls (median: 16 μg/m^3^).

**Table 3:** PM_10_ and NO_2_ concentrations in the homes of children classified as PTB cases and controls in the study

n	PM_10_, μg/m^3^		NO_2_, μg/m^3^	
	cases	controls	cases	controls
	107	127	107	127
GM (95% CI)	52 (47–57)	50 (46–54)	16 (15–17)	16 (15–17)
Min	6.6	12	6.0	4.5
25%	37	41	12	12
Median	48	41	15	16
75%	76	64	22	20
Max	173	223	45	55

Pollutant concentrations versus child, maternal and household characteristics are shown in Table 4. Concentrations were higher in informal and crowded homes, in households with mixed and dirty cooking fuels, with SHS and in households without opening windows. PM_10_ concentrations were also higher in the homes of those cases in which the caregiver had lower levels of education. None of the pollutant concentrations were statistically significant between cases and controls.

**Table 4:** Average concentrations of pollutants (μg/m^3^) in households of cases and controls with different characteristics

		PM_10_ concentrations		Mean NO_2_ concentrations	
		median (range) (μg/m^3^)		median (range) (μg/m^3^)	
		cases (*n* = 107)	controls (*n* = 127)	cases (*n* = 107)	controls (*n* = 127)
Child Characteristics					
Age					
	> 2 to ≤15	41 (21–118))	41 (12–119)	14 (7.0–45)	17 (9.4–26)
	≤2	49 (6.6–173)	47 (27–223)	19 (6.0–34)	16 (4.5–55)
Sex					
	Female	48 (18–173)	41 (13–223)	15 (6.0–32)	16 (4.5–30)
	Male	49 (6.6–148)	41 (12–119)	15 (8.2–45)	16 (9.4–55)
HIV positive**					
	No	43 (6.6–148)	41 (12–223)	15 (6.0–45)	16 (4.5–55)
	Yes	53 (26–173)	69 (31–105)	15 (7.5–34)	16 (9.4–26)
Household TB contact					
	No	48 (22–173)	41.0 (12–136)	14 (6.0–34)	15 (4.5–52)
	Yes	49 (6.6–149)	55 (35–223)	15 (7.0–45)	17 (9.4–55)
Maternal Characteristics					
Caregiver education					
	Post high school	37 (26–48)	41 (31–67)	12 (9.4–17)	15 (9.4–17.4)
	High school + below	51 (6.6–172)	41 (12–223)	15 (6.0–45)	16 (4.5–55)
Household Characteristics					
Type of housing structure					
	Formal	41 (6.6–118)	41 (12–223)	15 (6.0–34)	16 (4.5–55)
	Informal	76 (26–173)	76 (58–136)	22 (7.0–45)	21 (11–28)
Crowding					
	≤2	41 (18–148)	41 (12–223)	14 (6.0–35)	16 (4.5–52)
	> 2	65 (6.6–173)	76 (27–119)	17 (7.0–45)	17 (9.41–55)
Primary cooking fuel					
	Clean	43 (6.6–146)	41 (12–223)	15 (6.0–34)	16 (4.5–52)
	Mix + Dirty	88 (52–173)	108 (53–119)	21.56 (7.03–44.87)	23 (20–55)
Secondary cooking fuel					
	None + Clean	51 (21–173)	41 (12–223)	13 (6.0–45)	15 (9.4–28)
	Mix + Dirty	46 (6.6–146)	51 (13–136)	20 (9.0–35)	17 (4.5–55)
Secondhand smoke					
	No	48 (18–148)	41 (11–136)	14 (6.0–44	16 (4.5–55)
	Yes	48 (6.6–173)	64 (24–223)	16 (7.0–34)	16 (9.4–29)
Presence of opening windows					
	Some rooms	43 (6.6–148)	41 (12–223)	14.5 (6.0–45)	16 (4.5–55)
	No room	72 (37–172)	76 (27–136)	21 (8.4–35)	21 (11–30)
Burning of incense					
	No	48 (6.6–172)	41 (12–223)	18.0 (6.0–45)	16 (9.9–55)
	Yes	49 (21–114)	51 (13–136)	13 (7.5–29)	15 (4.5–44)
Visible surface water stains/dampness					
	No room	41 (18–119)	41 (13–119)	15 (6.0–35)	16 (9.4–44)
	Some rooms	51 (6.6–173)	48 (12–223)	15 (7.0–45)	16 (4.5–55)

Significantly different concentrations between households with different characteristics for both cases and controls are shown in bold

In the unadjusted analysis (Table 5), dampness (OR = 1.8, 95% CI: 1.01–3.1), cooking fuel type (OR = 2.6, 95% CI: 1.1–6.4), and SHS (OR = 1.7, 95% CI: 0.98–2.8), as well as PM_10_ concentrations (OR = 1.4, 95% CI: 0.8–2.3) were positively associated with PTB in children. When adjusted by other covariates, dampness, primary cooking fuel and secondhand smoke were still positively associated with an increased risk among cases, compared with controls, but with the exception of visible dampness (aOR = 2.4, 95% CI: 1.1–5.0), these were not statistically significant (Table 5). After adjusting for other variables, the risk of PTB was lower for increase in NO_2_ concentration (aOR = 0.4; 95% CI: 0.2–0.8) and not significantly associated with increase in PM_10_ (aOR = 0.9; 95% CI: 0.5–1.8).

**Table 5:** Crude and adjusted odds ratios for determinants of childhood PTB in different logistic models for indoor air environment variables

		Univariate	Multivariate models				
			Primary cooking fuel	Secondhand smoke	Dampness	PM_10_	NO_2_
Variable		OR (95% CI)	aOR (95% CI)	aOR (95% CI)	aOR (95% CI)	aOR (95% CI)	aOR (95% CI)
Primary cooking fuel	Clean	ref	ref				
	Mix + dirty	**2.6 (1.1–6.4)**	1.5 (0.5–4.6)				
^#^Secondhand smoke	No	ref		ref			
	Yes	1.7 (0.98–2.8)		1.2 (0.6–2.3)			
Visible dampness	No room	**ref**			**ref**		
	Some rooms	**1.8 (1.01–3.1)**			**2.4 (1.1–5.0)**		
PM_10_ concentrations	Median + below	ref				Ref	
	Above median	1.4 (0.8–2.3)				0.9 (0.5–1.8)	
NO_2_ concentrations	Median + below	ref					**ref**
	Above median	0.7 (0.4–1.2)					**0.4 (0.2–0.8)**
Age	≤2	**ref**	**ref**	**ref**	**ref**	**ref**	**ref**
	> 2–14	**0.3 (0.1–0.7)**	**0.2 (0.1–0.6)**	**0.2 (0.1–0.6)**	**0.2 (0.1–0.6)**	**0.2 (0.1–0.6)**	**0.2 (0.1–0.5)**
Sex	Female	ref	ref	ref	ref	ref	ref
	Male	1.3 (0.8–2.2)	1.2 (0.6–2.3)	1.2 (0.6–2.4)	1.3 (0.7–2.5)	1.2 (0.6–2.4)	1.3 (0.7–2.4)
**HIV + ve status	No	**ref**	**ref**	**ref**	**ref**	**ref**	**ref**
	Yes	**22 (7.4–63)**	**26 (8.4–83)**	**27 (8.6–84)**	**32 (9.7–104)**	**27 (8.7–87)**	**29 (9.0–93)**
Household TB contact	No	**ref**	**ref**	**ref**	**ref**	**ref**	**ref**
	Yes	**5.1 (2.9–9.0)**	**6.2 (3.1–12)**	**6.3 (3.2–12)**	**6.2 (3.1–12)**	**6.5 (3.3–13)**	**7.8 (3.8–16)**
Pseudo R^2^			0.31	032	0.33	0.31	0.33

All multivariate models were adjusted for age and sex. All models included crowding, HIV positive status of the child, presence of household TB contact and caregiver education

^#^Secondhand smoke assessed as presence of a smoker in the household

**Univariate model for HIV status results was based on those that had tested (*n* = 143). The missing observations were substituted as negative before inclusion to multivariate models

*OR* odds ratio

*aOR* adjusted odds ratio

There was a significant statistical association between risk factors for PTB disease: positive HIV status among children (ORs ranging from 21.5 to 31.8); younger age (ORs 0.2 to 0.3) and presence of a TB contact in the household (ORs 5.1 to 7.8) across all exposure models (Table 5).

## Discussion

In this, the first study to investigate associations between objective measures of indoor air pollution and childhood PTB, we did not find a significantly increased risk of acquiring childhood PTB with increasing PM_10_ and NO_2_ concentrations. Unexpectedly dampness, secondhand smoke exposure and use of dirty cooking fuels in the households were positively associated with acquiring PTB in childhood.

Some studies have described positive associations between PTB and type of fuel used for cooking for biomass [15–17]; and for kerosene [18]. However these findings were not replicated in other studies [19–21]. Although the “dirty fuels” risk among cases was increased compared to controls in our study, the majority of homes using “dirty fuel” were using kerosene. The effects of chronic exposure to kerosene smoke at a population level are not extensively studied but toxicity from exposure in animal and laboratory experiments is well-documented [22]. In the review of kerosene hazards by Lam et al. [22], the toxic effects of high levels of kerosene vapour and aerosols, and combustion products on neurological and pulmonary changes (bronchoconstriction, hyperirritability and inflammation) have been confirmed [22].

Secondhand smoke has been associated with either latent TB infection or active disease across different studies [5, 6, 23]. Different studies assessed exposure of children to SHS differently and therefore conclusions may differ depending on the accuracy of the methods. We report an increased risk when children are exposed to SHS measured as number of smokers living the children at home.

Dampness on the household surfaces as a determinant of PTB in children was an interesting finding in our study. Dampness has being implicated in fungal respiratory infections [24, 25] but no study has found an association with PTB. In 1900, after observations of different families living in damp houses and other studies on tuberculosis, Newton [26] argued that there could be a link between exposure to dampness and tuberculosis. The explanation for this could be that dampness is associated with indoor mould growth. Mould is known to aggravate respiratory distress or colonise cavities that are a result of TB lesion in the lungs [27]. Therefore exposure to mould or dampness indicates exposure to mycotoxins, glucans and volatile organic compounds (VOCs) that compromise immunity of the respiratory system. Mycotoxin has an effect on defense mechanisms that control the mycobacterium in the lungs. Some mycotoxins reduce the functioning of ciliary function, alveolar macrophages and acquired immunity of the lungs which is a key factor in reducing the entry and/or proliferation of mycobacterium in the lungs [28].

Although there is growing literature investigating the association between PTB and exposure to air pollution [6, 8, 16, 29–31], the findings have been inconsistent. We did not find any statistically significant association between indoor PM_10_ or NO_2_ concentrations and childhood PTB. In urban environments, indoor PM_10_ and NO_2_ is the result of a multitude of sources including outdoor sources such as traffic emissions and other outdoor pollutants [32–34]. This is in contrast to rural areas where cooking fuel is the major contributor [35–37]. In this study, 10 and 40% of households that were using non-clean sources of energy for cooking had elevated PM_10_ and NO_2_ concentrations respectively [14]. However, because of the high day-to-day variability of PM_10_ and NO_2_ concentrations observed within households, a single measurement is likely to result in misclassification of exposure and drive a possible association with health effects towards the null.

Chemical constituents in the particulate matter (PM) have been demonstrated to be an important aspect when linking exposure to health effects [38–41]. Speciation of collected PM_10_ might have shown some associations with active PTB because constituents of PM such as metals and polycyclic aromatic compounds (PAHs) are known to cause respiratory distress and/or compromise lung immunity towards infections (including TB) [42–44].

Household TB contact and HIV positive status are well-known risk factors for active and latent TB and these findings were confirmed in our study. Both in high and low TB burden countries, household TB contact, especially sputum positive cases, have been shown to be an important risk factors for TB transmission [45–47]. Our population of interest has a high HIV burden and it was not surprising that we found that almost 50% of the PTB cases were HIV positive. Using multivariable models, we conducted sensitivity analysis by first excluding controls that were never tested for HIV, and then included them as HIV negative in the analysis, but there was no substantial change in the risk estimates. The assumption that all controls not tested for HIV were HIV negative is based on the SA DoH guidelines [48]. Only highly exposed infants (mothers who are HIV positive) are tested for HIV at birth and later. Only when the child has signs of diseases, such as TB, is the HIV test done [48]. According to the South African official statistics, the prevalence rate of HIV in children in 2016 was 2.4% as compared to 12.6% in the adult population [49].

The strength of this study is that exposure to indoor air pollution was measured, and using a well standardized household walkthrough instrument, we were able to model homes not sampled. This approach has not been adopted in previous studies examining associations with PTB. Using a study design with children as participants, minimized the confounding factors encountered when studying adults such as smoking, alcohol intake and occupational exposures. Diagnosis of PTB in children using sensitive and specific GeneXpert was also a strength of this study as this minimised the risk of misclassification of the PTB status.

Estimating exposure to indoor air pollution in epidemiological studies is always a challenge especially in the resource-constrained environment. In this study, we only measured or estimated one 24-h measurement of PM_10_ concentration in each home to estimate exposure of the children. Although this approach of characterizing exposure is better than no measurements at all, the variability within homes (between days) may be higher than between homes [14]. If so, a single 24-h measurement may result in misclassification of exposure to indoor air pollution. The participants’ households of cases may have changed the practices when the children were diagnosed with PTB therefore the observed measured concentrations could be lower than prior the children were diseased.

## Conclusions

There was a positive association between SHS and type of cooking fuel used (kerosene) and active PTB disease, although not statistically significant in adjusted estimates. We found no increased risk associated with active PTB in children and measured increased PM_10_ and NO_2_ concentrations. Although this is the first study that used actual measurements to assess indoor air pollution exposure, we noted that reliable exposure assessment is nevertheless challenging due to high variability. In addition to well-known risk factors, such as household TB contact and HIV status, this study also suggested that indoor dampness is a risk factor for PTB in children.

## Abbreviations

DoH: Department of Health
HIV: Human immune deficiency syndrome
KZN: KwaZulu-Natal
NHLS: National Health Laboratory Services
NO_2_: Nitrogen oxide
OR: Odds Ratio
PM: Particulate matter
PTB: Pulmonary tuberculosis
SHS: Secondhand smoke
SO_2_: Sulphur dioxide
Stats SA: Statistics South Africa
